# Lactic Acid Bacteria in Chinese Sauerkraut: Its Isolation and *In Vitro* Probiotic Properties

**DOI:** 10.3390/foods14152690

**Published:** 2025-07-30

**Authors:** Ming-Yang Han, Wen-Yong Lou, Meng-Fan Li

**Affiliations:** 1 Food Science School, Guangdong Pharmaceutical University, Zhongshan 528458, China; 2 Lab of Applied Biocatalysis, School of Food Science and Engineering, South China University of Technology, Guangzhou 510641, China

**Keywords:** probiotic identification, *L. plantarum*, hyperuricemia, genomic analysis

## Abstract

Probiotics have been widely explored for their potential in managing hyperuricemia. However, their isolation and identification are fundamental prerequisites for practical application. In this study, 254 lactic acid bacteria (LAB) strains were isolated from Chinese sauerkraut and screened for probiotic potential based on genomic and phenotypic characteristics, as well as nucleoside-degrading activity relevant to decrease serum urate. Among them, *Lactiplantibacillus plantarum* (*L. plantarum*) F42 exhibited the highest bile salt tolerance (survivor rate: 19.46 ± 4.33%), strong adhesion to Caco-2 cells (1.89 ± 0.12%), effective nucleoside degradation (inosine: 5.46 ± 0.67 mg∙L^−1^∙min^−1^; guanosine: 3.84 ± 0.11 mg∙L^−1^∙min^−1^), and notable anti-listeria activity (inhibition zone: 6.9 ± 0.3 mm). Based on its functional profile, *L. plantarum* F42 was selected as a promising probiotic candidate for further investigation of its urate-lowering effects. This work provides a new insight into anti-hyperuricemia probiotic selection based on *in vitro* nucleoside-degrading activity.

## 1. Introduction

Hyperuricemia (HUA) has become a major public health concern worldwide due to its increasing prevalence and incidence [1]. Notably, this increasing prevalence contributes to the growing cost of urate-lowering and associated analgesic therapies [2]. Another major concern is the occurrence of serious adverse effects due to drug treatments, such as subclinical hypothyroidism [3] and kidney function impairment [4] induced by the administration of the urate-lowering medicine allopurinol, as well as an increased risk of cardiovascular death caused by supplementation with another urate-lowering medicine, febuxostat [5]. In contrast, probiotic-based interventions have recently attracted attention as a potential alternative strategy for managing HUA, owing to their promising safety and efficacy profiles.

Increasing evidence highlights the beneficial effects of probiotics, especially LAB in HUA management. For example, *Lactobacillus gasseri* PA-3, isolated from human feces, has been found to reduce serum urate levels in rats by inhibiting the absorption of purine-based compounds derived from dietary sources, as demonstrated in both *in vitro* [6] and *in vivo* studies [7]. Similarly, the *Lacticaseibacillus rhamnosus* Fmb14 strain has been shown to utilize dietary inosine as an energy source and engage in the production of folic acid as well as riboflavin, which probably results in a decrease in inosine uptake in HUA mice, thus demonstrating a suppressive effect on urate synthesis [8]. In addition, numerous *in vitro* studies have shown that various *Lactobacillus* strains have the potential to act as functional probiotics against HUA [9,10].

In accordance with the definition of ‘probiotics’ by the WHO, the main criteria for identifying a probiotic candidate include its tolerance to acid and bile salts, adhesive ability to gut epithelial cells, antagonistic activity against pathogens, and overall safety profile [11]. Moreover, ability to degrade nucleosides, such as inosine and guanosine, has been widely regarded as a supplemental criterion for selecting probiotic candidates with the potential function of attenuating HUA, supported by the promising correlation between its ability *in vitro* and decrease in serum urate levels *in vivo* [9,10,12]. Although the majority of probiotic strains are known to colonize the colon, studies have identified viable *Lactobacillus* species in the jejunum where nucleoside absorption predominantly occurs. This suggests that oral *Lactobacillus* strains with acid and bile tolerance may reach and remain active in the small intestine long enough to exert nucleoside-degrading activity before dietary nucleosides are absorbed into the bloodstream, thereby reducing the systemic purine load and subsequent uric acid production.

Despite growing interest in probiotic supplementation for HUA, few studies have systematically screened probiotics from traditional fermented foods based on their nucleoside-degrading activity. Moreover, comprehensive genomic and phenotypic evaluations of such strains remain limited. This study aimed to isolate a specific probiotic candidate with a high potential to attenuate HUA. Nucleoside degradation was used as an index to isolate LAB from Chinese sauerkraut. Subsequently, their genomic feature and probiotic properties—such as resistance to bile salts and low pH, potential colonization in the intestine, bacteriostatic ability, and safety evaluation—were compared to select one strain of *Lactobacillus* with great potential as a probiotic for further investigation of its function and mechanism in attenuating HUA.

## 2. Materials and Methods

### 2.1. Materials

Chinese sauerkraut, a traditional fermented food using Chinese cabbage as raw material, was provided by local market (Guangzhou, China). De Man–Rogosa–Sharpe (MRS), Luria–Bertani (LB) broth, and Columbia blood agar plate were purchased from Huankai Microbial Sci & Tech Co., Ltd. (Guangzhou, China). *Escherichia coli* (*E. coli*) ATCC 25922, *Staphylococcus aureus* (*S. aureus*) ATCC 51650, *Listeria monocytogenes* (*L. monocytogenes*) CMCC 54002, *Salmonella enterica* (*S. enterica*) GIM 1.237 and Caco-2 cells were purchased from the Guangdong Microbial Culture Center (Guangzhou, China). Basic media for human cell culture, such as DMEM, penicillin–streptomycin, trypsin, and fetal bovine serum (FBS), were purchased from Gibo Co., Ltd. (New York, NY, USA). Inosine and guanosine (purity > 99%) were provided by Macklin Co., Ltd. (Shanghai, China). All other chemicals used were of analytical grade or higher quality.

### 2.2. Isolation of LAB from Chinese Sauerkraut

One milliliter of Chinese sauerkraut juice was mixed with 9 mL of saline (0.85% *w/v* NaCl) for dilution ten times. Similarly, the appropriate decimal dilutions were performed. MRS agar containing 0.1% *w/v* bromocresol purple powder was employed as growth medium of LAB. Both pour and spread plate methods were used for LAB isolation; however, the spread plate method was selected for LAB enumeration. After 48 h of incubation at 37 °C, each colony surrounded by a yellow circle in the agar was streaked on the surface of a new MRS agar for second isolation. A single colony was then inoculated in a new MRS broth for microbial proliferation. Isolated LAB was collected in sterile tubes with a final concentration of glycerol of 25% *v/v* and stored at −80 °C until use.

### 2.3. Degradation of Nucleosides

Nucleoside degradation was measured using the method described by Yamada *et al.* [6] with minor modifications. Each isolated LAB culture was incubated in MRS broth at 37 °C for 18 h. Then, 1 mL of cell culture was collected, and MRS was replaced with the same volume of sodium phosphate buffer (0.1 M, pH 7.4) containing 1.3 mM inosine and 1.3 mM guanosine for a 1 h reaction at 37 °C. Subsequently, 0.25 mL of 5% *v/v* TFA was added to terminate the degradation reaction. Centrifugation was performed to collect the supernatant, which was then filtered through a 0.22 μm membrane filter for further quantification by high-performance liquid chromatography (HPLC) equipped with a detection wavelength of 254 nm, an Agilent ZORBAX SB-C18 column (Agilent, Santa Clara, CA, USA) (4.6 × 250 mm 5-Micron), and a mobile phase of 25 mM KH_2_SO_4_ buffer containing 5% *v/v* methanol. Nucleoside concentration was calculated using standard curves (inosine: *C* = 2.2 × 10^7^ A + 39,065, R^2^ = 0.999; guanosine: *C* = 2.5 × 10^7^ A + 34,183, R^2^ = 0.999). Degradation ability (DA) and degradation rate (V) were calculated according to the following equations:

(1) DA (%) = (C_0_ − C_1_)/C_0_ × 100

(2) V (mg∙L^−1^∙min^−1^) = (C_0_ − C_1_)/60 × 1000

where C_0_ and C_1_ indicate the concentrations of inosine and guanosine (mmol/L) before and after the reaction, respectively.

### 2.4. Strain Identification

The isolated colony was dispersed in 20 µL of 20 mmol/L NaOH solution, followed by a heating process at 99 °C for 15 min to release the DNA from cells. After centrifugation, the supernatant was collected as a DNA template for gene cloning via PCR reaction. The 16S rRNA and pheS genes were amplified using the following primers: 5′-agagtttgatcctggctcag-3′ for 27F; 5′-tacgacttaaccccaatcgc-3′ for 1492R. 5′-tactatgcaaacaagggtggtaccg-3′ for pheS-F; 5′-caacggccgtccgttcaatcttttc-3′ for pheS-R. The molecular weight of the PCR product was visualized by agarose electrophoresis, and the DNA sequence was confirmed by Sangon Company (Shanghai, China). The DNA sequence was subjected to a BLAST (2.17.0) search against the NCBI nucleotide database, and species were assigned based on the criteria that both sequence similarity and sequence coverage with reference sequences exceeded 90%.

### 2.5. Genome Sequencing Analysis

The total genomic DNA of LAB was extracted strictly in accordance with the manufacturer’s instructions provided with the Bacterial Genomic DNA Extraction Kit (Sangon, Shanghai, China). DNA was fragmented into approximately 500 base pairs in length using an ultrasonic DNA disruptor, following the operation parameters provided by the manufacturer. A DNA library was then constructed according to the Hieff NGS^®^ MaxUp II DNA Library Prep Kit for Illumina^®^ (Sangon, Shanghai, China). DNA sequences of LAB were obtained using the MGI DNBSEQ-T7 platform. Raw reads were subjected to quality control using Fastp (0.11.2). Specifically, reads were discarded if they contained ≥ 40% low-quality bases or if the trimmed length of paired-end reads was <35 nt. After filtering, the average read depth of the LAB genome reached 150 bp, and the average length of the predicted genes was 860 bp. Sequence similarity searches were performed using the BLASTn tool (2.2.28) against the NCBI NT database, and results were retained if the sequence identity > 90% and the coverage > 80%. Gene function was annotated using multiple databases. In particular, the basic metabolisms of carbohydrates, amino acids, and nucleotides were analyzed based on their pathways obtained from the KEGG and COG databases.

### 2.6. Growth Curves

*Lactobacillus* was inoculated in MRS broth at a concentration of 1% *v*/*v*. Biomass was monitored every 2 h of anaerobic incubation on the first day and 24 h on the next three days. Bacterial cultures were centrifugated at 12,000 rpm for 1 min, and the supernatant was replaced with an equal volume of saline. The biomass was identified as the UV absorbance at 600 nm with saline used as a blank control.

### 2.7. Resistance to Bile Salts and Low pH

The growth of *Lactobacillus* as a function of salt concentration and pH was evaluated according to a previous study with modifications [13]. *Lactobacillus* (1% *v*/*v*) was inoculated in MRS broth containing different concentrations of bile salts (0, 0.03 and 0.3%, *w*/*v*.) or under different pH values (3.0 and 7.0). After 2 h of incubation, the number of viable *Lactobacillus* was determined using the pour plate method. The percentage survival rate was expressed as the number of viable cells incubated in harsh conditions (bile salts or low pH) against the viable counts incubated in pure MRS.

### 2.8. Colonization in Intestine

The colonization ability of *Lactobacillus* was evaluated based on chemical and cellular assessments. Auto-aggregation, coaggregation, and hydrophobicity indexes were determined following the method reported by Tuo *et al.* [14] to evaluate the potential colonization ability of Lactobacillus. For cellular assessment, Caco-2 cells (1 × 105 cells per well) were inoculated and incubated at 37 °C in a 5% CO_2_ atmosphere for 6 days. During incubation, the fresh DMEM medium in the presence of 20% *v*/*v* bovine serum and 1% *v*/*v* penicillin–streptomycin was used to replace the old one every two days. After that, the *Lactobacillus* pellets, obtained by centrifugation and washing twice with HBSS, were resuspended in fresh HBSS and added to the Caco-2 wells. After 2 h of incubation, free *Lactobacillus* was removed through four washes with HBSS. Then, the number of viable cells adhering to the Caco-2 cell line was determined using the pour plate method. Adhesion ability was calculated as follows:

(3) Adhesion ability (%) = N_t_/N_0_ × 100

where N_t_ and N_0_ represent the viable count of *Lactobacillus* adhering to Caco-2 cells and the total viable count of inoculated *Lactobacillus*, respectively.

### 2.9. Bacteriostatic Ability

The well diffusion agar method was employed to measure the antibacterial activity, as described in a previous study [15]. LB agar was melted and cooled to 48 °C. Then 0.1% *v/v* of the intestinal pathogen including Gram-negative (*E. coli* and *S. enterica*) and Gram-positive (*S. aureus* and *L. monocytogenes*) strains was mixed with LB agar. Wells with 8 mm diameter were made in LB–agar plates containing 0.1% *v/v E. coli*, *S. enterica*, *S. aureus* or *L. monocytogenes*, and 100 μL of *Lactobacillus* supernatant was added to each well. After overnight incubation at 37 °C, the diffusion diameter was recorded. The antibacterial zone was equal to the diffusion zone minus 8 mm.

### 2.10. Antibiotic Susceptibility

The agar disk diffusion method was used to assess the antibiotic susceptibility of the isolated *Lactobacillus* strains, as described previously [11]. Three groups of antibiotics were tested, including the inhibitors of nucleic acid synthesis (5 μg of ciprofloxacin and rifampicin), protein synthesis (30 μg chloramphenicol, kanamycin, tetracycline and 15 μg erythromycin), and cell wall synthesis (30 μg vancomycin, amoxicillin, and 10 μg ampicillin). Results were expressed in terms of resistant (R), intermediately sensitive (I), and sensitive (S) levels.

### 2.11. Hemolysis

Hemolytic activity was evaluated to assess the safety of the bacteria, as described previously [16]. In brief, *Lactobacillus* was inoculated onto a Columbia blood agar plate and defibrillated sheep blood. *S. aureus* was used as positive control. After an incubation at 37 °C for 24 h, the grow halos around the colonies were recorded and expressed in terms of γ-hemolysis (no halo), α-hemolysis (greenish halos), and β-hemolysis (bright halos). They were classified as non-hemolytic, partially hemolytic, and hemolytic.

### 2.12. Statistical Analysis

All experiments were performed in triplicate. Data are shown as mean ± s.d. (standard deviation) and were visualized using Prism GraphPad 8.3.0 software. The multiple-group comparisons were carried out through a one-way ANOVA analysis followed by Tukey’s honestly significant difference (HSD) post hoc test for pairwise comparisons. *p* < 0.05 was considered statistically significant, as indicated by * or various letters between groups.

## 3. Results and Discussion

### 3.1. LAB Diversity in Chinese Sauerkraut

**Figure 1:**
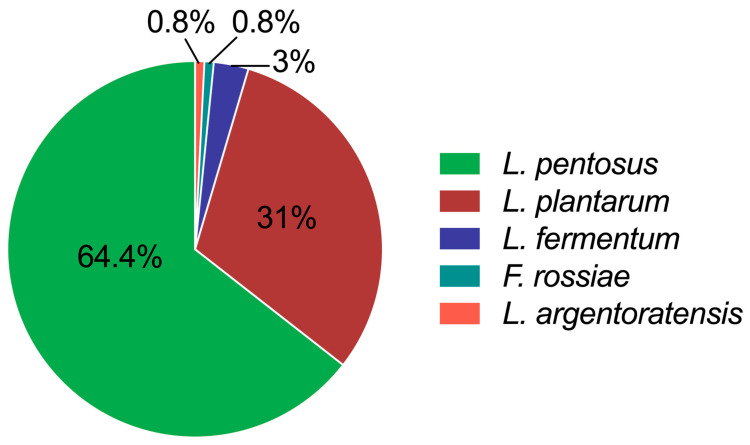
Diversity of LAB in 50-day-fermented sauerkraut collected from Guangzhou markets. Data were calculated as the isolation frequencies of the species or subspecies relative to the total number of identified isolates.

As shown in Figure 1, all identified isolates belonged to four species—*Lactiplantibacillus pentosus* (*L. pentosus*), *L. plantarum*, *Limosilactobacillus fermentum* (*L. fermentum*), and *Furfurilactobacillus rossiae* (*F. rossiae*)—and one subspecies of *L. plantarum*, *L. plantarum* subsp. *argentoratensis* (*L. argentoratensis*). Their abundances were 64%, 31%, 3%, 0.8% and 0.8%, respectively. The results indicated that the predominant LAB species in Chinese sauerkraut are *L. pentosus* and *L. plantarum* species, which is in agreement with the literature on fermented mustard, beet, and eggplant [17]. *L. pentosus* was often inaccurately referred to as *L. plantarum* due to its high similarity in physiological (e.g., shape, growth conditions, and metabolic properties) or partial genetic traits (i.e., the conserved region of the 16S rRNA gene) [18]. Some studies have demonstrated that *L. plantarum* species have a higher abundance than *L. pentosus* or that only *L. plantarum* species are present in fermented vegetables [19,20,21]. This difference could be explained by the fact that the microbial composition and population of traditional fermented foods differ by geographic position, materials, and fermentation stages [22]. However, *Lactobacillus* sp. is the dominant LAB genus that plays essential roles in the fermentation of carbohydrates [23] and contributes to food’s organolepticity, quality, and safety [24].

*L. pentosus*, *L. plantarum,* and *L. fermentum* belonging to *Lactobacillus* sp. have been frequently found in various native fermented vegetables. In particular, *L. plantarum* and *L. fermentum* species have been approved as common edible probiotics by China’s National Health Commission. Their wide range of applications have been explored in the food industry as natural preservatives and texture and flavor enhancers [25]. Furthermore, they can be found as active ingredients in probiotic supplements or functional foods due to their promising health benefits, e.g., enhancing gut health by maintaining a balanced intestinal microbiota, improving digestion, and modulating immunity [26]. Although *L. pentosus* has not yet been officially approved as a probiotic in China, its potential beneficial effects on host health—such as the regulation of gut microbiota [27], protection against viral infection, and attenuation of alcoholic liver injury—have attracted considerable attention [28]. *F. rossiae* species, as an obligately hetero-fermentative LAB like *L. fermentum* but unlike *L. plantarum*, were scarcely found in traditional fermented vegetables [29]. However, *F. rossiae* has been reported as a starter culture of cocoa fermentation to produce D-L lactic acid by utilizing glucose and fructose, leading to the resistance of cocoa-related stress conditions (e.g., low pH, high temperature, and high osmotic pressure) [30]. *L. argentoratensis* is a subspecies of the *L. plantarum* species; however, its functionality and applications in the food industry have rarely been studied.

### 3.2. Nucleoside-Degrading Activity of LAB

*Lactobacillus* species exhibit species-specific differences in their ability to degrade nucleosides [31]. As a result, the top fifty strains isolated from Chinese sauerkraut exhibited various abilities and rates of degradation for inosine and guanosine (Table S1). Notably, nine strains of LAB were capable of complete degradation of inosine and guanosine (Table 1), and they are as follows: *L. plantarum* F42, *L. pentosus* P38, *L. plantarum* 44, *L. argentoratensis* 15, *L. fermentum* S8641, *L. pentosus* b21, *L. pentosus* S856s, *L. fermentum* SI823, and *L. plantarum* b8643. In contrast, no nucleoside-degrading ability was found in *L. casei* and *L. paracasei*, which were both isolated from commercial dairy products. Previous studies have reported that the nucleoside-degrading reaction is probably catalyzed by the intracellular nucleoside N-ribohydrolase for RNA and DNA synthesis and energy supply for cellular processes, whereby the enzyme is widely present in LAB, including *L. plantarum* [9], *L. brevis* [32], *L. gasseri* [33] and *L. fermentum* [34]. Here, four different strains of LAB (*L. plantarum* F42, *L. pentosus* P38, *L. argentoratensis* 15, and *L. fermentum* S8641) with the highest degradation ability for nucleosides were selected for further evaluation of their probiotic properties based on genotypic and phenotypic traits.

### 3.3. Genomic Characterization of LAB

#### 3.3.1. General Genomic Features

The GC content of the genome of all *Lactobacillus* species is in the range of 43–51% (Table 2), which is in accordance with the *Lactobacillus* genus [29,32]. The number of annotated genes in *Lactobacillus* was consistently over 3000, except for the *L. fermentum* S8641, which contained only 1859 genes. This could imply that genomic research on *L. fermentum* is relatively less extensive or that its annotation has not reached a depth comparable to that of other *Lactobacillus* species [35]. The percentage of annotated genes in *Lactobacillus* differed between databases (Figure S1). Due to the high homology between *L. plantarum* and *L. argentoratensis*, their percentage of annotated genes in each database was nearly identical. In comparison, *L. pentosus* exhibited the highest percentage of genes annotated by CDD, PFAM, GO and KEGG database, suggesting that a considerable level of depth has been achieved in genomic research on this strain. Overall, a substantial number of annotated genes in all *Lactobacillus* were found to be associated with carbohydrate and amino acid metabolism pathways (Figures S2 and S3).

#### 3.3.2. Carbohydrate Metabolism

**Figure 2:**
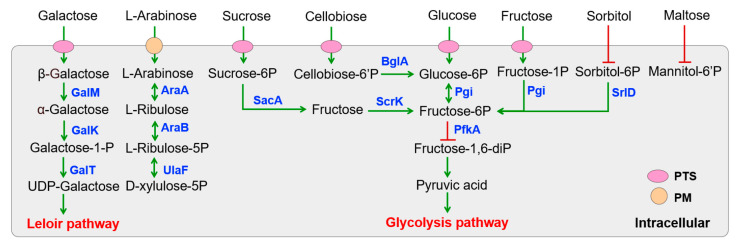
KEGG pathway-based sugar metabolism in *Lactobacillus* isolated from Chinese sauerkraut. PTS (phosphotransferase systems) and PM (plasma membrane transporter) are involved in sugar transport. Genes encoded enzymes are shown in blue. Red lines with bars indicate a potential missing reaction in *L. fermentum* S8641.

**Figure 3:**
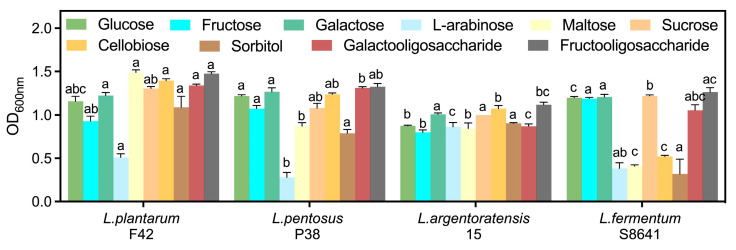
Growth activity (OD_600nm_) of *Lactobacillus* in MRS broth with glucose replaced by different carbohydrates. Different letters indicate significant difference (*p* < 0.05) between *Lactobacillus* in the same medium.

The genome sequence analysis revealed a substantial number of genes involved in PEP-PTS systems, ABC transporters, and permeases for carbohydrate utilization in various *Lactobacillus* strains (Table S2). Specifically, all *Lactobacillus* strains displayed conserved carbohydrate specificity predictions, with the exception of *L. fermentum* S8641, which notably lacked sorbitol recognition and maltose transport capabilities (Figure 2). This gene deficiency resulted in poor growth and proliferation when sorbitol or maltose served as the sole carbon source (Figure 3). Despite the absence of the pfkA gene encoding 6-phosphofructokinase, *L. fermentum* S8641 demonstrated robust growth when sucrose, glucose, or fructose was supplied as the sole carbon source. This phenotype could be attributed to its phosphoketolase (PK) pathway, in which hexoses are cleaved, ultimately generating CO_2_, ethanol, acetate, and lactate as fermentation end-products [36]. Conversely, *L. plantarum* F42, *L. pentosus* 38, and *L. argentoratensis* 15 were capable of utilizing maltose and sorbitol. Moreover, their metabolism of glucose, sucrose, and fructose was conducted via the glycolysis pathway. This highlights the metabolic versatility within the genus, where different strains employ distinct pathways to adapt to varying carbohydrate substrates. In addition, all *Lactobacillus* could utilize cellobiose, galactooligosaccharide, and fructooligosaccharide for proliferation due to the presence of genes encoding a series of enzymes (e.g., α-amylase, α-galactosidase, β-galactosidase genes cluster, Endo-beta-N-acetylglucosaminidase D, glycosyl hydrolases) which are responsible for the degradation of di-, tri-, and even polysaccharides [37].

#### 3.3.3. Amino Acid Metabolism

**Figure 4:**
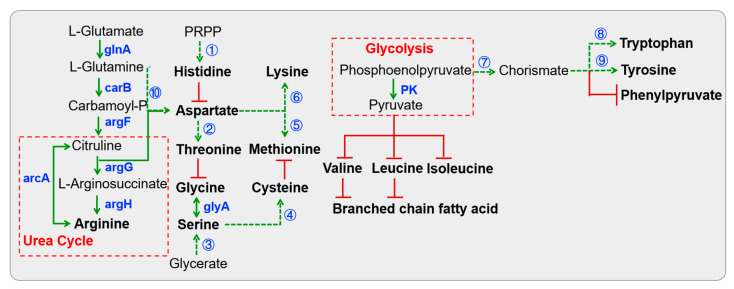
KEGG pathway-based amino acid biosynthesis in *Lactobacillus* isolated from Chinese sauerkraut. Red lines with bars indicate a potential missing reaction. Dotted arrows indicate a multi-step reaction. The first multi-step reaction is catalyzed by a series of *his* enzymes including *hisA*, *hisB*, *hisC*, *hisD*, *hisE*, *hisF*, *hisG*, *hisI* and *hisZ*. The second multi-step reaction is catalyzed by *lysC*, *asd*, *thrB1* and *thrC*. The third multi-step reaction is catalyzed by *garK*, *gpmA*, *serA* and *serC*. Enzymes *cysE* and *cysK* participate in the fourth reaction. The fifth reaction is catalyzed by *lysC*, *asd*, *metA*, *metB*, *metC* and *mmuM*. Numerous enzymes (*lysA*, *lysC*, *asd*, *dapA*, *dapB*, *dapE*, *dapF*, *dapG*, *dapH* and *mtnE*) are involved in the sixth reaction. Enzymes *aroA*, *aroB*, *aroC*, *aroD*, *aroE*, *aroK* and *EC:2.5.1.19* participate in the seventh reaction. Enzymes *trpA*, *trpB*, *trpC*, *trpD*, *trpE*, *trpF* and *trpG* participate in the eighth reaction. Enzymes *EC:5.4.99.5*, *tyrA2* and *hisC* participate in the ninth reaction, and the tenth reaction is catalyzed by the three enzymes (*carA*, *caiB* and *pyrB*).

All *Lactobacillus* strains putatively dedicated the *glnA* enzyme to the conversion of glutamate into glutamine (Figure 4 and Table S3), which is associated with the beginning of arginine biosynthesis and glutamate degradation. Furthermore, previous studies have evidenced that the non-essential amino acid had positive effects on the intestinal mucosal system in the host [37]. Additionally, a series of *arg* and *car* enzymes potentially converted glutamate into aspartate, which ultimately led to the formation of arginine. Glycerate could be converted into serine through a series of metabolic reactions. Serine then serves as a precursor for the synthesis of glycine and cysteine. However, the pathway for cysteine synthesis from serine may be absent in *L. fermentum* S8641 due to the lack of the enzyme serine O-acetyltransferase (*cysE*). Additionally, conversion of threonine into glycine was not universal among *Lactobacillus* species. Nevertheless, they possessed an almost complete pathway for generating histidine from PRPP, which involves the pentose phosphate pathway. Conversely, only *L. fermentum* S8641 was capable of synthesizing tyrosine as it possessed the enzyme *EC 5.4.99.5*. This metabolic diversity highlights the species-specific metabolic capabilities and limitations within the genus, underscoring the genetic and biochemical adaptations that enable these bacteria to synthesize essential amino acids.

#### 3.3.4. Nucleotide Metabolism

**Figure 5:**
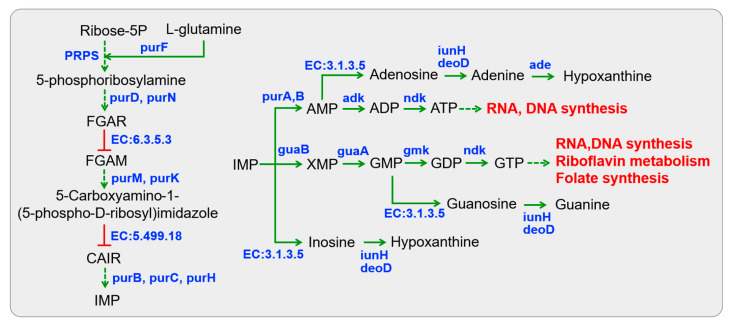
KEGG pathway-based nucleotide metabolism in *Lactobacillus* isolated from Chinese sauerkraut. Red lines with bars indicate a potential missing reaction in all *Lactobacillus* species.

The first step in purine metabolism involves de novo purine biosynthesis, which initiates with a phosphorylation reaction of ribose-5-phosphate and culminates in the formation of IMP (inosine monophosphate) (Figure 5). All *Lactobacillus* putatively possessed the metabolic capability to engage in the de novo purine biosynthesis (Table S4). However, they were unable to autonomously complete a series of reactions in de novo purine biosynthesis due to the absence of two essential enzymes (phosphoribosylformylglycinamidine synthase, EC:6.3.5.3; 5-(carboxyamino) imidazole ribonucleotide mutase, EC:5.499.18).

IMP acts as a substrate for the subsequent synthesis of AMP (adenosine monophosphate) and GMP (guanosine monophosphate). All *Lactobacillus* were predicted to be capable of participating in ATP and GTP metabolism through reversible conversions from AMP and GMP, respectively, represented by enzymes adenylate kinase (*adk*, EC:2.7.4.3), nucleoside-diphosphate kinase (*ndk*, EC:2.7.4.6), and guanylate kinase (*gmk*, EC:2.7.4.8). This process predominantly provides energy for cellular activities, and the products can be utilized in RNA and DNA synthesis, riboflavin metabolism, and folate biosynthesis. Only *L. pentosus* P38 was capable of synthesizing nucleosides (e.g., inosine, guanosine, and adenosine) from IMP, GMP, and AMP through dephosphorylation (5′-nucleotidase, EC:3.1.3.5). For nucleoside degradation, two specific enzyme-encoding genes (purine nucleosidase, *iunH*, EC:3.2.2.1; adenine deaminase, *ade*, EC:3.5.4.2) were present in all *Lactobacillus*, which convert nucleosides into purine bases such as inosine to hypoxanthine, guanosine to guanine, and adenosine to adenine and hypoxanthine. By comparison, the presence of an isoenzyme urine-nucleoside phosphorylase (*deoD*, EC:2.4.2.1) was a peculiarity of *L. fermentum* S8641, leading to nucleoside degradation. This observation was in accordance with the abovementioned results, which indicated a strong ability to degrade inosine and guanosine in all tested *Lactobacillus* strains (Table 1). Finally, these nucleosides and their degraded products could be metabolized as the substrates of DNA synthesis or participate in the metabolism of clycine, serine, and threonine.

Overall, the metabolic pathways of four *Lactobacillus* strains were characterized by the identification of gene presence based on genotypic analysis. However, these genotype-based results are susceptible to potential artifacts from gene annotation and the genome assembly processes. Therefore, phenotypic validation, such as targeted metabolic profiling, would be critical in future work to confirm the metabolic pathways in these strains.

### 3.4. Phenotypic Characterization of LAB

#### 3.4.1. Growth Curves

**Figure 6:**
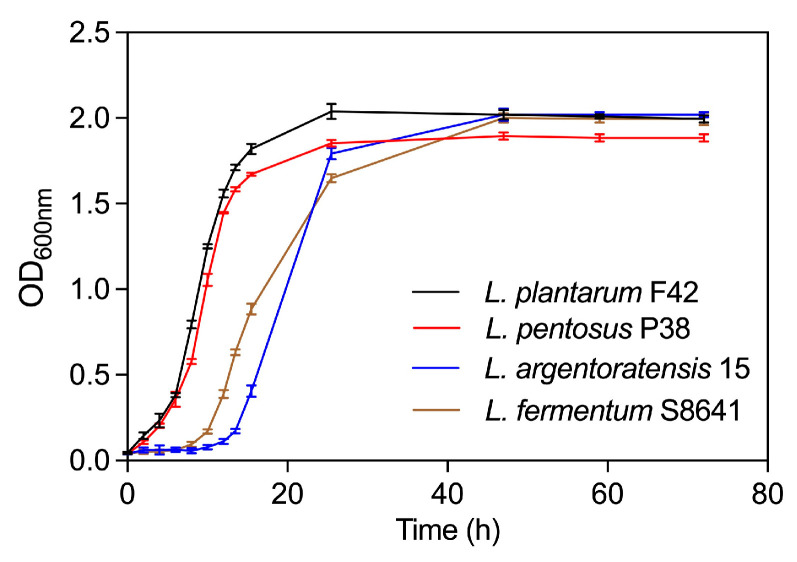
Growth curves of four *Lactobacillus* species derived from Chinese sauerkraut.

As shown in Figure 6, *L. plantarum* F42 exhibited a comparable pattern of growth kinetics to *L. pentosus* P38, with equivalent periods allocated for the lag phase (from 0 to 2 h), logarithmic (log) growth phase (at 4 h until 16 h), and stationary phase (after 24 h). This strongly suggests that both bacterial species had similar temporal dynamics during their respective growth cycles under identical or closely matched environmental conditions. In addition, the lag phase duration of *L. argentoratensis* 15 was found to be longer than that of *L. fermentum* S8641. Specifically, the lag phase for *L. argentoratensis* 15 extended from 0 to 12 h, whereas *L. fermentum* S8641 exhibited a lag phase lasting from 0 to 8 h. This indicates a difference in their initial adaptation periods before entering exponential growth. Finally, both species started the stationary phase between 24 and 48 h of cultivation after undergoing a different duration of log phase. Overall, the precise timing for entering each growth phase was probably variable in different *Lactobacillus* strains due to their specific growth requirements and metabolic capabilities.

#### 3.4.2. Resistance to Bile Salts and Low pH

The resistance of LAB to bile salts and low pH during the simulated digestion is a pivotal criterion for assessing their potential as probiotic candidates. As shown in Table 3, the initial viable count of all tested *Lactobacillus* was in the range of 6.79–7.19 log CFU mL^−1^, with no significant differences between strains (*p* > 0.05). After 2 h of incubation in a simulated environment with a low pH level of 3.0, which represented postprandial human stomach acidity, a significant decrease in the survival rate of all tested *Lactobacillus* strains to 61.79–77.51% was observed. Specifically, *L. plantarum* F42 (77.51 ± 1.29%) and *L. argentoratensis* 15 (72.47 ± 1.72%) exhibited a significantly higher survival rate than *L. pentosus* P38 (66.78 ± 3.26%, *p* < 0.05) and *L. fermentum* S8641 (61.79 ± 1.25%, *p* < 0.05).

In addition, the viability of all *Lactobacillus* significantly decreased in the presence of 0.03% bile salts, which mimicked the small intestine at its lowest concentration of bile salts. Finally, the survival rate was in the following order: *L. plantarum* F42 > *L. argentoratensis* 15 > *L. pentosus* P38 > *L. fermentum* S8641. The decrease in cell viability underscores the fact that low pH and bile salts threaten *Lactobacillus*. Furthermore, numerous studies have consistently demonstrated a reduction in the viability of different *Lactobacillus* strain under harsh gastrointestinal conditions, but with strain-dependent resistance or sensitivity [38,39]. By comparison, *L. plantarum* SF4722 exhibited a higher survival rate than the other three strains under such conditions (*p* < 0.05), indicating its potential to remain alive until reaching the intestine, where it can exert beneficial effects on the host’s health. Rzepkowska et al. reported that the differences in the resistance of LAB to bile salts could be attributed to their innate bile salt hydrolase, which deconjugates bile salts [40]. According to the genomic features, a gene encoding the specific enzyme bile salt hydrolase (EC:3.5.1.24), which is involved in bile acid biosynthesis, was found in all tested *Lactobacillus*, and the presence of this gene was in the following order, from highest to lowest: *L. plantarum* F42 (8) = *L. argentoratensis* 15 (8) > *L. pentosus* P38 (6) > *L. fermentum* S8641 (4). However, the hypothesis that the correlation between the quantity of bile salt hydrolase/genes in *Lactobacillus* and its resistance to bile salts requires further investigation.

#### 3.4.3. Potential Colonization in the Intestine

**Figure 7:**
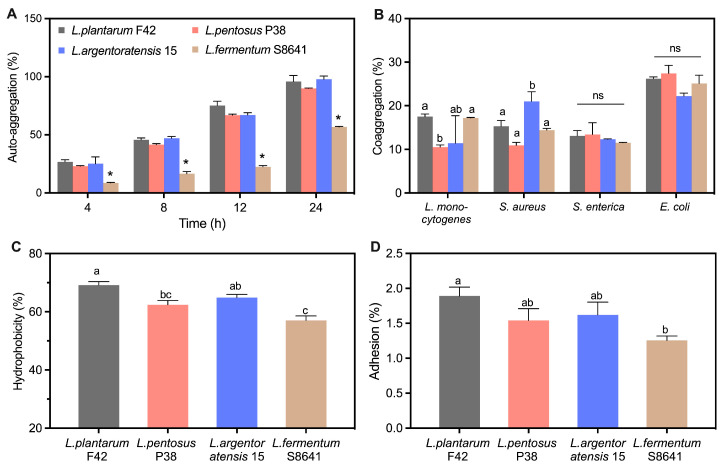

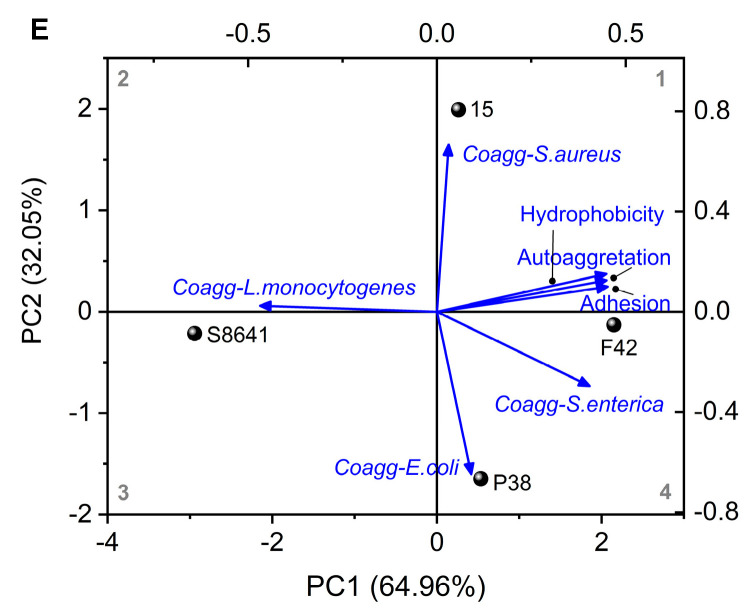
Auto-aggregation (**A**), coaggregation with four pathogens (*L. monocytogenes*, *S. aureus*, *S. enterica* and *E. coli*) (**B**), hydrophobicity (**C**), adhesion ability to Caco-2 cells (**D**), and the principal component analysis (PCA) (**E**) integrating the results of auto-aggregation, coaggregation, hydrophobicity and adhesion ability of *Lactobacillus* derived from Chinese sauerkraut. Vectors represent trait loadings; longer vectors indicate stronger contributions to principal components (PC1: 64.96% variance, PC2: 32.05% variance). Different letters indicate significant difference (*p* < 0.05) between groups. *: *p* < 0.05.

All *Lactobacillus* strains presented an auto-aggregation capacity ranging from 8.7 to 26.8% after 4 h incubation (Figure 7A), where the *L. fermentum* S8641 strain exhibited the lowest value, but *L. plantarum* F42 displayed the highest value. The auto-aggregation capacity of *L. fermentum* S8641 at 8.7% is consistent with the literature showing the auto-aggregation percentage of eleven strains of *L. fermentum* was in the range of 3.5 to 14.2% under similar incubation conditions [13]. In addition, the auto-aggregation of all tested *Lactobacillus* gradually increased by incubation time. Notably, no significant differences were detected among *L. plantarum* F42, *L. pentosus* P38, and *L. argentoratensis* 15 at each incubation time; however, their auto-aggregation values were significantly higher than that of *L. fermentum* S8641. Previous studies have illustrated that bacterial aggregation ability, including auto- and co-aggregation, is potentially linked to their adhesion to intestinal epithelial cells, which in turn affects their survival and persistence within the gastrointestinal tract [14]. All tested strains had a greater coaggregation with *E. coli* compared to the other three pathogenic species (Figure 7B), which is in accordance with previous publications [39,41].

The hydrophobicity of the bacterial surface has been recognized as an important factor in promoting their ability to adhere to host tissues [14]. This attachment mechanism is often mediated by the affinity between the hydrophobic regions of bacterial cell surfaces and the similar regions on host tissue cells or mucus layers lining the gastrointestinal tract. The hydrophobicity was also strain-specific (Figure 7C). The mean value of hydrophobicity for all tested strains followed the order *L. plantarum* F42 > *L. argentoratensis* 15 > *L. pentosus* P38 > *L. fermentum* S8641, with the highest value for *L. plantarum* F42 (69.2%) and the lowest value for *L. fermentum* S8641 (57.0%). However, a similar trend to that observed in hydrophobicity was found in the adhesion properties of these tested strains (Figure 7D). *L. plantarum* F42 showed the highest adhesion percentage compared to other three strains, which is consistent with previous studies showing that the adhesion of *L. plantarum* species to Caco-2 cell line was higher than *L. fermentum* species [41]. The components on the surface of bacterial cell walls play an important role in the adhesive properties they exhibited. Tuo et al. summarized that the surface-bound protein of bacteria contributes to its adhesive properties, as indicated by the fact that the adhesion of *Lactobacillus* to Caco-2 cells was significantly decreased after treating with protein denaturant [14]. Lebeer et al. illustrated that the surface proteins and mucin-binding proteins on probiotics play a critical role in their interaction with the intestinal mucus layer [42]. Specifically, the Leu-Pro-any-Thr-Gly sequence at the C-terminus of mucin-binding proteins can link to peptidoglycan in the cell wall, functioning as a bridge between the probiotic and gut mucus layer, ultimately facilitating bacterial adhesion and colonization [43]. Furthermore, bacterial adhesion characteristics could also be attributed to the glycoproteins and teichoic and lipoteichoic acids present on the surface of the bacterial cell wall [44].

The results obtained from the assays (regarding the hydrophobicity, auto-aggregation, and coaggregation ability of *Lactobacillus* with various pathogens) were subjected to PCA analysis to identify the potential correlations between these bacterial characteristics and their adhesive properties. As shown in Figure 7E, the first two principal components (PC1: 64.96%; PC2: 32.05%), which accounted for 97.01% of the overall variance, were sufficient for further discussion (*p* < 0.05). The circle points representing the four tested *Lactobacillus* strains were distributed separately in different quadrants, indicating a diversity of bacterial properties among them. The vectors displayed bacterial characteristics. Notably, the hydrophobicity and auto-aggregation abilities were close to the adhesion properties. As shown in Table 4, their positive loading values on PC1 (> 0.44) were larger than coaggregation with different pathogens (−0.469–0.404). These results indicate a strong positive correlation between both traits and adhesion ability. In addition, the point *L. plantarum* F42 was closer to the adhesion properties than other points/strains, implying its superior potential to adhere to the intestine.

#### 3.4.4. Bacteriostatic Ability

As shown in Table 5, different strains of *Lactobacillus* displayed distinct inhibitory capacities against various pathogens. *L. plantarum* F42, *L. argentoratensis* 15, and *L. fermentum* S8641 exhibited antibacterial activity against the four tested pathogens, with a low inhibition against *E.coli* (+) and a moderate inhibition against *S. aureus* (++). This result is consistent with previous publications showing that *Lactobacillus* strains were more active against Gram-positive bacteria [40]. Antibacterial activity against *S. aureus* was not found in *L. pentosus* P38, although inhibition of the other three pathogens occurred. Notably, *L. plantarum* F42 had stronger anti-listeria (*L. monocytogenes*) activity than other *Lactobacillus* strains. Previous studies have demonstrated that *Lactobacillus* exhibits antibacterial activity against a variety of pathogenic microorganisms (e.g., *L. monocytogenes*, *S. aureus*, *S. enterica*, and *E.coli*), which can be attributed to their competitive adhesion to the epithelium cells or to the secretion of antibacterial substances (e.g., organic acids, bacteriocin, and hydrogen peroxide) [11,45]. It has been evidenced that bacteriocin production is a general feature of *Lactobacillus*. Bacteriocins, especially the class IIa type, have strong inhibitory activity against *L. monocytogenes* strains [40]. *L. plantarum* strains are known to produce a diversity of bacteriocins, which could induce membrane leakage and cell death by specifically binding the mannose phosphotransferase system (man-PTS) on their target pathogenic cells [46]. According to the genetic features, genes encoding two specific enzymes (*LciA* and *LagD*) which involve the formation and transport of bacteriocin were found in *L. plantarum* F42 and *L. argentoratensis* 15 strains. In particular, the presence of genes encoding a variety of plantaricin systems (*plnE*, *plnF*, *plnJ* and *plnK*) putatively contributed to the production of at least two types of bacteriocins. However, those genes were not detected in the *L. fermentum* S8641 strain, implying that the low-pH environment resulting from its organic acid is a crucial factor in inhibiting pathogen growth.

#### 3.4.5. Antibiotic Susceptibility

All tested strains were resistant to the ciprofloxacin, an inhibitor of nucleic acid synthesis (Table 6), which is in accordance with previous publications showing the resistance of *L. fermentum*, *L. plantarum*, and its subsp. strains to ciprofloxacin [9,11]. The tested strains exhibited various degrees of sensitivity to rifampicin, with *L. plantarum* F42 and *L. argentoratensis* 15 showing resistance, while *L. pentosus* P38 and *L. fermentum* S8641 demonstrated intermediate sensitivity. All *Lactobacillus* were susceptible to chloramphenicol and tetracycline, the inhibitors of protein synthesis, while none of the strains were susceptible to kanamycin. All tested strains were intermediately susceptible to erythromycin except for *L. fermentum* S8641, which was resistant to erythromycin. Similar observations have been demonstrated by previous studies [16,47].

In addition, resistance to the inhibitors of cell wall synthesis, vancomycin and amoxicillin, was found in all tested strains; however, the strains were consistently susceptible to ampicillin. Inherent non-acquired resistance to vancomycin in a variety of *Lactobacillus* species has been consistently reported [45]. Overall, numerous studies have shown that most probiotics are naturally resistant to ciprofloxacin, kanamycin, and vancomycin; however, these resistances are suspected to encode within their chromosomes and are neither inducible nor horizontally transferable among bacteria [48,49]. Although no probiotic health claims related to antibiotic resistance have been positively assessed by the European Food and Safety Agency (EFSA), *Lactobacillus* species still can be recommended for inclusion in the qualified presumption of safety (QPS) list [50] as they have a long history of safe use and have never been implicated in human or animal diseases.

#### 3.4.6. Safety Evaluation

According to WHO and EFSA guidelines, the absence of hemolytic activity is a fundamental criterion for selecting probiotic strains to guarantee their non-virulent nature. Therefore, hemolytic activity was employed to evaluate the safety of isolated *Lactobacillus* strains for applications as bio-preservatives or probiotics in food industry. As a result, the positive control *S. aureus* exhibited β-hemolytic activity (Table 7). In contrast, all tested *Lactobacillus* strains were classified as γ-hemolytic, demonstrating that they are non-hemolytic and therefore safe for use.

## 4. Conclusions

In this study, *Lactobacillus* species including *L. pentosus*, *L. plantarum*, *L. fermentum* and *L. argentoratensis* were successfully isolated from Chinese sauerkraut. These strains exhibited different nucleoside-degrading activities, which are potentially associated with urate-lowering effects in vivo. Four different strains (*L. plantarum* F42, *L. pentosus* P38, *L. argentoratensis* 15, and *L. fermentum* S8641) demonstrated complete degradation of inosine and guanosine (100%) and were selected for further evaluation of probiotic potential. Genomic analysis demonstrated their similar metabolic pathways related to carbohydrates, amino acids and nucleotides, except for *L. fermentum* S8641. Among them, *L. plantarum* F42 showed higher tolerance to low pH (survival rate: 77.51 ± 1.29%) and bile salts (survival rate: 19.46 ± 4.33%). PCA analysis illustrated that the adhesion ability (PC1 loading = 0.452) of *Lactobacillus* is positively correlated with its surface hydrophobicity (0.449) and auto-aggregation abilities (0.449), supporting the superior gut colonization potential of *L. plantarum* F42 (1.89 ± 0.12%). Furthermore, *L. plantarum* F42 displayed broad antimicrobial activity against both Gram-positive (*L. monocytogenes*: 6.9 ± 0.3 mm; *S. aureus*: 4.4 ± 0.5 mm) and Gram-negative bacteria (*S. enterica*: 4.2 ± 0.3 mm, *E. coli*: 1.9 ± 0.1 mm). Its safety for use in the food industry was further supported by the lack of hemolytic activity on blood agar plates. Considering these advantageous characteristics, *L. plantarum* F42 was identified as a promising probiotic candidate for anti-hyperuricemia. Moreover, the strain could be incorporated into fermented foods (e.g., yogurt, probiotic drinks) or as a therapeutic candidate for individuals at risk of hyperuricemia. This study might open up opportunities to develop targeted probiotic isolation strategies aimed at managing hyperuricemia, although their in vivo anti-hyperuricemia effects should be confirmed using animal and clinical tests in the future in order to advance their application in the fields of functional foods and therapeutics.

## Figures and Tables

**Table 1:** The nucleoside-degrading ability of nine LAB strains.

Strains	DA_Ino_ (%)	*V*_Ino_ (mg∙L^−1^∙min^−1^)	DA_Guo_ (%)	*V*_Guo_ (mg∙L^−1^∙min^−1^)
*L. plantarum* F42	100 ± 1.41	5.46 ± 0.67 ^a^	100 ± 1.27	3.84 ± 0.11 ^a^
*L. pentosus* P38	100 ± 2.82	5.40 ± 0.40 ^a^	100 ± 6.90	3.81 ± 0.24 ^a^
*L. plantarum* 44	100 ± 5.66	5.32 ± 0.58 ^a^	100 ± 5.36	3.80 ± 0.17 ^a^
*L. argentoratensis* 15	100 ± 4.66	5.31 ± 0.43 ^a^	100 ± 11.41	3.80 ± 0.75 ^a^
*L. fermentum* S8641	100 ± 2.55	3.78 ± 0.47 ^b^	100 ± 3.11	2.52 ± 0.55 ^b^
*L. pentosus* b21	100 ± 0.85	3.78 ± 0.17 ^b^	100 ± 2.07	2.52 ± 0.13 ^b^
*L. pentosus* S856s	100 ± 7.92	3.78 ± 0.50 ^b^	100 ± 3.11	2.52 ± 0.18 ^b^
*L. fermentum* Sl823	100 ± 9.48	3.78 ± 0.58 ^b^	100 ± 1.84	2.51 ± 0.18 ^b^
*L. plantarum* b8643	100 ± 7.64	3.78 ± 0.49 ^b^	100 ± 0.71	2.51 ± 0.08 ^c^

DA_Ino_ and DA_Guo_ represent inosine and guanosine degradation ability, respectively; *V*_Ino_ and *V*_Guo_ denote their degradation rates. Different superscript letters within the same column indicate significant difference (*p* < 0.05) between strains.

**Table 2:** General genome features of *Lactobacillus*.

Feature	*L. plantarum* F42	*L. pentosus* P38	*L. argentoratensis* 15	*L. fermentum* S8641
Total reads count	7,538,286	10,170,832	8,320,090	8,982,324
Genome size	3,422,275	3,723,671	3,326,539	1,904,611
Average read length	149.28	145.26	149.00	149.19
GC content	44.06%	47.08%	43.45%	51.33%
Clean reads	99.90%	99.90%	99.91%	99.91%
Total genes	3235	3525	3099	1859

**Table 3:** The viability (log CFU mL^−1^) and survival rate of four *Lactobacillus* species at low pH (3.0) and in the presence of 0.03% porcine bile salts (BS) at pH 7.0.

Strains	Initial Viability	pH3.0		0.03% BS at pH7.0	
		Viability	Survival Rate	Viability	Survival Rate
*L. plantarum* F42	7.19 ± 0.30 ^a^	7.08 ± 0.29 ^a^	77.51 ± 1.29% ^a^	6.47 ± 0.20 ^a^	19.46 ± 4.33% ^a^
*L. pentosus* P38	6.81 ± 0.10 ^a^	6.63 ± 0.08 ^a^	66.78 ± 3.26% ^b^	5.85 ± 0.22 ^ab^	11.14 ± 3.03% ^b^
*L. argentoratensis* 15	6.79 ± 0.16 ^a^	6.65 ± 0.15 ^a^	72.47 ± 1.72% ^a^	6.00 ± 0.14 ^ab^	16.18 ± 0.79% ^b^
*L. fermentum* S8641	6.97 ± 0.16 ^a^	6.76 ± 0.16 ^a^	61.79 ± 1.25% ^b^	5.71 ± 0.05 ^b^	5.68 ± 1.52% ^b^

Different superior letters within the same column indicate significant difference (*p* < 0.05) between strains.

**Table 4:** Trait loadings on PC1 and PC2.

Traits	PC1 Loading	PC2 Loading
Hydrophobicity	0.449	0.151
Auto-aggregation	0.449	0.125
Adhesion ability	0.452	0.100
Coagg-*S. enterica*	0.404	−0.296
Coagg-*S. aureus*	0.031	0.666
Coagg-*E. coli*	0.091	−0.648
Coagg-*L. monocytogenes*	−0.469	0.024

A larger absolute value suggests that the variable significantly influences the component.

**Table 5:** Antimicrobial activity of four *Lactobacillus* species against four pathogens.

Strains	*L. monocytogenes*	*S. aureus*	*S. enterica*	*E. coli*
*L. plantarum* F42	6.9 ± 0.3 ^a^ (+++)	4.4 ± 0.5 ^a^ (++)	4.2 ± 0.3 ^a^ (++)	1.9 ± 0.1 ^a^ (+)
*L. pentosus* P38	1.7 ± 0.3 ^b^ (+)	0.0 ± 0.1 ^b^ (−)	4.7 ± 1.3 ^a^ (++)	4.4 ± 0.8 ^b^ (++)
*L. argentoratensis* 15	3.1 ± 0.1 ^c^ (++)	2.9 ± 0.4 ^c^ (++)	1.6 ± 0.2 ^b^ (+)	1.3 ± 0.3 ^a^ (+)
*L. fermentum* S8641	3.6 ± 0.5 ^c^ (++)	2.6 ± 0.4 ^c^ (++)	3.0 ± 0.5 ^a^ (++)	0.9 ± 0.4 ^a^ (+)

−: ≤0 mm; +: 0–2 mm; ++: 2–5 mm; +++: >5 mm. Different superscript letters within the same column indicate significant difference (*p* < 0.05) between strains.

**Table 6:** Antibiotic susceptibility of four *Lactobacillus* species.

Groups	Antibiotics	*L. plantarum* F42	*L. pentosus* P38	*L. argentoratensis* 15	*L. fermentum* S8641
I	Ciprofloxacin	R	R	R	R
	Rifampicin	R	I	R	I
II	Chloramphenicol	S	I	S	I
	Kanamycin	R	R	R	R
	Erythromycin	I	I	I	R
	Tetracycline	S	I	S	S
III	Vancomycin	R	R	R	R
	Amoxicillin	R	R	R	R
	Ampicillin	S	S	S	S

R: resistant; I: intermediately sensitive; S: sensitive. Group I: inhibitors of nucleic acid synthesis; Group II: inhibitors of protein synthesis; Group III: inhibitors of cell wall synthesis.

**Table 7:** Hemolytic activity of four *Lactobacillus* species.

Strains	Hemolytic Activity
*S. aureus*	β-hemolysis
*L. plantarum* F42	γ-hemolysis
*L. pentosus* P38	γ-hemolysis
*L. argentoratensis* 15	γ-hemolysis
*L. fermentum* S8641	γ-hemolysis

β-hemolysis: hemolytic; γ-hemolysis: non-hemolytic.

## Data Availability

The original contributions presented in this study are included in the article. Further inquiries can be directed to the corresponding authors.

## Supplementary Materials

The following supporting information can be downloaded at: https://www.mdpi.com/article/10.3390/foods14152690/s1, Table S1: The top fifty strains of LAB ranked by the ability to degrade nucleosides; Table S2: The number of genes and their predicted specificity for carbohydrate transport and metabolism in various *Lactobacillus*; Table S3: Putative classification of amino acid metabolism; Table S4: Putative classification of nucleotide metabolism; Figure S1: Percentage of annotated genes in different databases; Figure S2: KEGG pathway annotated gene categories for different *Lactobacillus* species; Figure S3: COG classification of *Lactobacillus*.
