# Supplementary material for: Lactic Acid Bacteria in Chinese Sauerkraut: Its Isolation and In Vitro Probiotic Properties
Source: Foods. 2025 Jul 30;14(15):2690. doi: 10.3390/foods14152690 (PMC12346754; doi:10.3390/foods14152690)
Supplement: Supplementary file 1 [file foods-14-02690-s001.zip › foods-3778048-supplementary.pdf]

foods

Supplementary materials

Lactic acid bacteria in fermented vegetables: its isolation and *in vitro* probiotic properties

Ming-Yang Han[1], Wen-Yong Lou[2]*, Meng-Fan Li[1]*

[1] Food Science School, Guangdong Pharmaceutical University, Zhongshan 528458, China

[2] Lab of Applied Biocatalysis, School of Food Science and Engineering, South China University of Technology, Guangzhou 510641, China

* Corresponding author

Email address: wylou@scut.edu.cn; limengfan@gdpu.edu.cn

Table S1. The top fifty strains of LAB ranked by the ability to degrade nucleosides. DA_{Ino} and DA_{Guo} indicates the degradation ability for inosine and guanosine, respectively. V_{Ino} and V_{Guo} means the degradation rate toward inosine and guanosine, respectively.

Strain	DA_{Ino} (%)	V_{Ino} (g·L^{-1}·min^{-1})	DA_{Guo} (%)	V_{Guo} (g·L^{-1}·min^{-1})	Identification
F42	100	5.4E-03	100	3.8E-03	*Lactiplantibac*[illegible]
P38	100	5.4E-03	100	3.8E-03	*Lactiplantibac*[illegible]
44	100	5.3E-03	100	3.8E-03	*Lactiplantibac*[illegible]
15	100	5.3E-03	100	3.8E-03	*L. plantarum s*[illegible]
S8641	100	3.78E-03	100	2.52E-03	*Limosilactoba*[illegible]
b21	100	3.78E-03	100	2.52E-03	*Lactiplantibac*[illegible]
S856s	100	3.78E-03	100	2.52E-03	*Lactiplantibac*[illegible]
Sl823	100	3.78E-03	100	2.51E-03	*Limosilactoba*[illegible]
b8643	100	3.78E-03	100	2.51E-03	*Lactiplantibac*[illegible]
23	99	3.72E-03	100	2.50E-03	*Lactiplantibac*[illegible]
21	73	2.79E-03	85	2.15E-03	*Lactiplantibac*[illegible]
LB3	70	2.66E-03	80	2.04E-03	*Lactiplantibac*[illegible]
s8645b	68	2.60E-03	81	2.06E-03	*Lactiplantibac*[illegible]
bb1noa	65	2.45E-03	79	2.00E-03	*Lactiplantibac*[illegible]
s71	60	2.26E-03	75	1.89E-03	*Lactiplantibac*[illegible]
S2	55	2.08E-03	58	1.46E-03	*Lactiplantibac*[illegible]
FS583	48	1.81E-03	57	1.43E-03	*Lactiplantibac*[illegible]

Strain	DA_{Ino} (%)	V_{Ino} ($g \cdot L^{-1} \cdot min^{-1}$)	DA_{Guo} (%)	V_{Guo} ($g \cdot L^{-1} \cdot min^{-1}$)	Identification
23	48	1.78E-03	56	1.41E-03	*Lactiplantibaci*
S853	48	1.78E-03	56	1.41E-03	*Furfurilactoba*
S741	46	1.72E-03	47	1.17E-03	*Lactiplantibaci*
P733	42	1.58E-03	43	1.08E-03	*Lactiplantibaci*
S731	41	1.55E-03	50	1.26E-03	*Lactiplantibaci*
C235	40	1.51E-03	49	1.24E-03	*Lactiplantibaci*
61	40	1.48E-03	41	1.03E-03	*Lactiplantibaci*

Strain	DA_{Ino} (%)	V_{Ino} ($g \cdot L^{-1} \cdot min^{-1}$)	DA_{Guo} (%)	V_{Guo} ($g \cdot L^{-1} \cdot min^{-1}$)	Identification
82	36	1.43E-03	39	1.05E-03	*Lactiplantibacillus plantarum*
81	35	1.31E-03	44	1.12E-03	*Lactiplantibacillus pentosus*
32	35	8.85E-04	39	1.53E-03	*Lactiplantibacillus pentosus*
62	34	1.28E-03	39	9.87E-04	*Lactiplantibacillus pentosus*
bb2	34	1.35E-03	37	9.97E-04	*Lactiplantibacillus pentosus*
P622	33	1.25E-03	41	1.04E-03	*Lactiplantibacillus pentosus*
P634	32	1.19E-03	39	9.87E-04	*Lactiplantibacillus pentosus*
P748	31	1.16E-03	33	8.38E-04	*Lactiplantibacillus pentosus*
L31	31	1.15E-03	35	8.79E-04	*Lactiplantibacillus pentosus*
s8642	29	1.09E-03	31	7.88E-04	*Lactiplantibacillus pentosus*
s844	29	6.68E-04	31	1.32E-03	*Lactiplantibacillus pentosus*
P732	27	1.2E-03	29	1.1E-03	*Lactiplantibacillus pentosus*
liub3	27	5.95E-04	26	1.19E-03	*Lactiplantibacillus pentosus*
S862	27	1.01E-03	31	7.79E-04	*Lactiplantibacillus pentosus*
P731	27	1.01E-03	36	9.17E-04	*Lactiplantibacillus pentosus*
Zhong41	27	1.01E-03	32	7.95E-04	*Lactiplantibacillus pentosus*
5128	27	5.89E-04	22	1.10E-03	*Lactiplantibacillus pentosus*
514	27	1.00E-03	28	7.04E-04	*Lactiplantibacillus pentosus*
5121	26	9.60E-04	26	6.55E-04	*Lactiplantibacillus pentosus*
1	26	5.42E-04	27	1.23E-03	*Lactiplantibacillus pentosus*
4	24	8.96E-04	25	6.24E-04	*Lactiplantibacillus pentosus*
30	23	8.75E-04	27	6.77E-04	*Lactiplantibacillus pentosus*
7	23	4.56E-04	21	1.06E-03	*Lactiplantibacillus pentosus*
5235	22	8.39E-04	25	6.21E-04	*Lactiplantibacillus pentosus*
5230	22	9.7E-04	22	8.7E-04	*Lactiplantibacillus pentosus*
Control group					
L. casei	1	5.5E-05	-2	-9.55E-05	*Lacticaseibacillus casei*
L. paracasei	-2	-1.12E-04	-0.5	-2.83E-05	*Lacticaseibacillus paracasei*

Table S2. The number of genes and its predicted specificity for carbohydrate transport and metabolism in various *Lactobacillus*. PEP-PTS means the phosphoenolpyruvate phospho-transferase systems, and ABC transporter indicates the ATP-binding cassette type transport.

Classification	*L. plantarum* F42	*L. pentosus* P38	*L. argentoratensis* 15
PEP-PTS	60	51	39

Classification	*L. plantarum* F42	*L. pentosus* P38	*L. argentoratensis* 15
	Cellobiose<br>Fructose<br>Sorbitol<br>Galactitol<br>Mannitol<br>Mannitol/fructose<br>Glucose/maltose/*N*-acetyl-glucosamine	Cellobiose<br>Fructose<br>Sorbitol<br>Galactitol<br>Mannitol<br>Mannitol/fructose<br>Glucose/maltose/*N*-acetyl-glucosamine	Cellobiose<br>Fructose<br>Sorbitol<br>Galactitol<br>Mannitol<br>Mannitol/fructose<br>Glucose/maltose/*N*-acetyl-glucosamine
ABC transporters	13	12	13
	Maltose<br>Ribose<br>Polyol<br>Polysaccharide	Maltose<br>Ribose	Maltose<br>Ribose<br>Polyol<br>Polysaccharide
Permeases	21	41	20
	Maltose<br>Polyol<br>Arabinose<br>Putative glucose<br>H^+/gluconate	Maltose<br>Arabinose<br>Fucose<br>Putative glucose	Maltose<br>Polyol<br>Arabinose<br>Putative glucose<br>H^+/gluconate
Extracellular or cell wall-related polysaccharide degradation	12	10	10
	α-galactosidase<br>β-galactosidase<br>α-amylase<br>Endo-beta-*N*-acetylglucosaminidase D	α-galactosidase<br>β-galactosidase<br>α-amylase<br>Endo-beta-*N*-acetylglucosaminidase D	α-galactosidase<br>β-galactosidase<br>α-amylase<br>Endo-beta-*N*-acetylglucosaminidase D

Table S2 (*continued*)

Classification	*L. plantarum* F42	*L. pentosus* P38	*L. argentoratensis* 15	*L. [illegible]*
Initial degradation for saccharides	1	1	1	--
	Glycosyl hydrolases (family 31)	Glycosyl hydrolases (family 31)	Glycosyl hydrolases (family 31)	--
Galactose metabolism	5	5	6	4
	Galactokinase (GalK)<br>Galactose-1-phosphate uridyltransferase<br>Galactose mutarotase and related enzymes	Galactokinase<br>Galactose-1-phosphate uridyltransferase<br>Galactose mutarotase and related enzymes	Galactokinase<br>Galactose-1-phosphate uridyltransferase<br>Galactose mutarotase and related enzymes	[illegible]
Arabinose metabolism	4	26	5	3
	L-arabinose isomerase<br>Ribulose-5-phosphate 4-epimerase<br>Arabinose efflux permease	L-arabinose isomerase<br>Ribulose-5-phosphate 4-epimerase<br>Arabinose efflux permease	L-arabinose isomerase<br>Ribulose-5-phosphate 4-epimerase<br>Arabinose efflux permease	[illegible]
Rhamnose metabolism	18	17	18	8
	L-rhamnosidase<br>3-O-α-glucopyranosyl-L-rhamnose phosphorylase	L-rhamnosidase<br>3-O-α-glucopyranosyl-L-rhamnose phosphorylase	L-rhamnosidase<br>3-O-α-glucopyranosyl-L-rhamnose phosphorylase	[illegible]

Classification	*L. plantarum* F42	*L. pentosus* P38	*L. argentoratensis* 15	*L. f*
	N-acetylglucosaminyl-PP-decaprenol α-1,3-L-rhamnosyltransferase	*N*-acetylglucosaminyl-PP-decaprenol α-1,3-L-rhamnosyltransferase	*N*-acetylglucosaminyl-PP-decaprenol α-1,3-L-rhamnosyltransferase	

Table S3. The putative classification on the amino acid metabolism. Enzymes were abbreviated and their information could be checked according to the EC number in KEGG database.

Classification	*L. plantarum* F42	*L. pentosus* P38	*L. argentoratensis* 15	*L. f*
Arginine biosynthesis	11<br>*glnA (EC:6.3.1.2),*<br>*(E1.1.1.3),*<br>*pmA (EC:5.4.2.11),*<br>*argG (EC:6.3.4.5),*<br>*argH (EC:4.3.2.1),*<br>*argB (EC:2.7.2.8),*<br>*argC (EC:2.3.1.35),*<br>*(EC:2.3.1.1),*<br>*argD (EC:2.6.1.11*<br>*2.6.1.17),*<br>*argF (EC:2.1.3.3),*<br>*argJ (EC:2.3.1.35 2.3.1.1)*	9<br>*glnA (EC:6.3.1.2),*<br>*pmA (EC:5.4.2.11),*<br>*argG (EC:6.3.4.5),*<br>*argH (EC:4.3.2.1),*<br>*argB (EC:2.7.2.8),*<br>*(EC:2.3.1.1),*<br>*argD (EC:2.6.1.11*<br>*2.6.1.17),*<br>*argF (EC:2.1.3.3),*<br>*argJ (EC:2.3.1.35 2.3.1.1)*	9<br>*glnA (EC:6.3.1.2),*<br>*argB (EC:2.7.2.8),*<br>*argC (EC:2.3.1.35),*<br>*(EC:2.3.1.1),*<br>*argD (EC:2.6.1.11),*<br>*(EC:2.6.1.17),*<br>*argF (EC:2.1.3.3),*<br>*argG (EC:6.3.4.5),*<br>*argH (EC:4.3.2.1),*<br>*argJ (EC:2.3.1.35 2.3.1.1)*	14<br>*gln*<br>*arg*<br>*arg*<br>*(EC*<br>*arg*<br>*(EC*<br>*arg*<br>*arg*<br>*arg*<br>*arg*<br>*arc*<br>*arc*<br>*roc*
Alanine, aspartate, and glutamate metabolism	24<br>*asnA (EC:3.5.1.1),*<br>*asnB (EC:6.3.5.4),*<br>*NIT2 (EC:3.5.1.3),*<br>*racD (EC:5.1.1.13),*<br>*gadB (EC:4.1.1.15),*<br>*glnA (EC:6.3.1.2),*<br>*gabD (EC:1.2.1.16*<br>*1.2.1.79 1.2.1.20),*<br>*aspA (EC:4.3.1.1),*<br>*carA (EC:6.3.5.5),*<br>*carB (EC:6.3.5.5),*<br>*glmS (EC:2.6.1.16),*<br>*purA (EC:6.3.4.4),*<br>*purB (EC:4.3.2.2),*<br>*patA (EC:2.6.1),*<br>*patB (EC:4.4.1.8),*<br>*ldh (EC:1.1.1.27), pyrB (EC:2.1.3.2)*	20<br>*NIT2 (EC:3.5.1.3),*<br>*aspA (EC:4.3.1.1),*<br>*asnA (EC:3.5.1.1),*<br>*asnB (EC:6.3.5.4),*<br>*glnA (EC:6.3.1.2),*<br>*gabD (EC:1.2.1.16*<br>*1.2.1.79 1.2.1.20),*<br>*aspA (EC:4.3.1.1),*<br>*carA (EC:6.3.5.5),*<br>*carB (EC:6.3.5.5),*<br>*glmS (EC:2.6.1.16),*<br>*purA (EC:6.3.4.4),*<br>*purB (EC:4.3.2.2),*<br>*patB (EC:4.4.1.8),*<br>*ldh (EC:1.1.1.27),*<br>*pyrB (EC:2.1.3.2)*	24<br>*asnA (EC:3.5.1.1),*<br>*asnB (EC:6.3.5.4),*<br>*NIT2 (EC:3.5.1.3),*<br>*racD (EC:5.1.1.13),*<br>*gadB (EC:4.1.1.15),*<br>*glnA (EC:6.3.1.2),*<br>*gabD (EC:1.2.1.16*<br>*1.2.1.79 1.2.1.20)*<br>*aspA (EC:4.3.1.1),*<br>*carA (EC:6.3.5.5),*<br>*carB (EC:6.3.5.5),*<br>*purA (EC:6.3.4.4),*<br>*purB (EC:4.3.2.2),*<br>*purF (EC:2.4.2.14),*<br>*glmS (EC:2.6.1.16),*<br>*pyrB (EC:2.1.3.2)*	23<br>*asn*<br>*asn*<br>*NIT*<br>*rac*<br>*gad*<br>*gln*<br>*gab*<br>*1.2.*<br>*asp*<br>*car*<br>*car*<br>*pur*<br>*pur*<br>*pur*<br>*glm*<br>*pyr*

Table S3 (*continued*)

Classification	*L. plantarum* F42	*L. pentosus* P38	*L. argentoratensis* 15	*L. f*
Glycine, serine and threonine metabolism	22<br>*lysC (EC:2.7.2.4),*<br>*asd (EC:1.2.1.11),*<br>*(EC:1.1.1.3),*<br>*thrB1 (EC:2.7.1.39),*<br>*thrC (EC:4.2.3.1),*<br>*glyA (EC:2.1.2.1),*<br>*serA (EC:1.1.1.95),*<br>*serC (EC:2.6.1.52),*<br>*gpmA (EC:5.4.2.11),*<br>*garK (EC:2.7.1.165),*<br>*adh (E1.1.1.1)*	18<br>*dsdA (EC:4.3.1.18),*<br>*lysC (EC:2.7.2.4),*<br>*asd (EC:1.2.1.11),*<br>*(EC:1.1.1.3),*<br>*thrB1 (EC:2.7.1.39),*<br>*thrC (EC:4.2.3.1),*<br>*glyA (EC:2.1.2.1),*<br>*serA (EC:1.1.1.95),*<br>*serC (EC:2.6.1.52),*<br>*gpmA (EC:5.4.2.11),*<br>*garK (EC:2.7.1.165)*	20<br>*lysC (EC:2.7.2.4),*<br>*asd (EC:1.2.1.11),*<br>*(EC:1.1.1.3),*<br>*thrB1 (EC:2.7.1.39),*<br>*thrC (EC:4.2.3.1),*<br>*glyA (EC:2.1.2.1),*<br>*serA (EC:1.1.1.95),*<br>*serC (EC:2.6.1.52),*<br>*gpmA (EC:5.4.2.11),*<br>*garK (EC:2.7.1.165),*<br>*sdaA (EC:4.3.1.17),*	20<br>*lys*<br>*asd*<br>*(EC*<br>*thr*<br>*thr*<br>*gly*<br>*ser*<br>*ser*<br>*gpn*<br>*gar*<br>*sda*

Classification	*L. plantarum* F42	*L. pentosus* P38	*L. argentoratensis* 15	*L. f*[illegible]
			lpd (EC:1.8.1.4),<br>*asd (EC:1.2.1.11),*<br>*asdA (EC:4.3.1.18)*	*lpd*[illegible]<br>*asd*[illegible]<br>*asd*[illegible]
Cysteine and methionine metabolism	31<br>*cysE (EC:2.3.1.30),*<br>*cysK (EC:2.5.1.47),*<br>*metC (EC:4.4.1.13)*<br>*(EC:1.1.1.3),*<br>*metE (EC:2.1.1.14),*<br>*lysC (EC:2.7.2.4),*<br>*mmuM (EC:2.1.1.10),*<br>*asd (EC:1.2.1.11),*<br>*metA (EC:2.3.1.46),*<br>*metB (EC:2.5.1.48),*<br>*luxS (EC:4.4.1.21),*<br>*mtnN (EC:3.2.2.9),*<br>*metY (EC:2.5.1.49)*	30<br>*cysE (EC:2.3.1.30),*<br>*cysK (EC:2.5.1.47),*<br>*(EC:1.1.1.3),*<br>*metE (EC:2.1.1.14),*<br>*lysC (EC:2.7.2.4),*<br>*mmuM (EC:2.1.1.10),*<br>*asd (EC:1.2.1.11),*<br>*metA (EC:2.3.1.46),*<br>*metB (EC:2.5.1.48),*<br>*luxS (EC:4.4.1.21),*<br>*mtnN (EC:3.2.2.9),*<br>*metY (EC:2.5.1.49)*<br>*mtnE (EC:2.6.1),*<br>*(E1.8.4.14)*	32<br>*cysE (EC:2.3.1.30),*<br>*cysK (EC:2.5.1.47),*<br>*metC (EC:4.4.1.13)*<br>*(EC:1.1.1.3),*<br>*metE (EC:2.1.1.14),*<br>*lysC (EC:2.7.2.4),*<br>*mmuM (EC:2.1.1.10),*<br>*asd (EC:1.2.1.11),*<br>*metA (EC:2.3.1.46),*<br>*metB (EC:2.5.1.48),*<br>*luxS (EC:4.4.1.21),*<br>*mtnE (EC:2.6.1),*<br>*gshA (EC:6.3.2.2),*<br>*patB (EC:4.4.1.8),*<br>*metY (EC:2.5.1.49)*	28<br>*cys*[illegible]<br>*met*[illegible]<br>*(EC*[illegible]<br>*lys*[illegible]<br>*mm*[illegible]<br>*asd*[illegible]<br>*met*[illegible]<br>*met*[illegible]<br>*lux*[illegible]<br>*mtn*[illegible]<br>*mtn*[illegible]<br>*met*[illegible]<br>*pat*[illegible]

Table S3 (*continued*)

Classification	*L. plantarum* F42	*L. pentosus* P38	*L. argentoratensis* 15	*L. f*[illegible]
Valine, leucine and isoleucine degradation/ biosynthesis	6<br>*ilvE (EC:2.6.1.42),*<br>*lpd (EC:1.8.1.4),*<br>*mmsB (EC:1.1.1.31),*<br>*(EC:2.3.3.10),*<br>*ilvB (EC:2.2.1.6)*	6<br>*ilvE (EC:2.6.1.42),*<br>*lpd (EC:1.8.1.4),*<br>*mmsB (EC:1.1.1.31),*<br>*(EC:2.3.3.10),*<br>*ilvB (EC:2.2.1.6)*	6<br>*ilvE (EC:2.6.1.42),*<br>*lpd (EC:1.8.1.4),*<br>*(EC:2.3.3.10),*<br>*mmsB (EC:1.1.1.31),*<br>*ilvB (EC:2.2.1.6)*	7<br>*ilv*[illegible]<br>*lpd*[illegible]<br>*(EC*[illegible]<br>*mm*[illegible]<br>*ato*[illegible]<br>*ilv*[illegible]
Lysine biosynthesis /degradation	21<br>*lysA (EC:4.1.1.20),*<br>*dapF (EC:5.1.1.7),*<br>*dapE (EC:3.5.1.18),*<br>*dapH (EC:2.3.1.89),*<br>*(EC:2.6.1.17),*<br>*(EC:3.5.1.47),*<br>*murE (EC:6.3.2.13),*<br>*murF (EC:6.3.2.10),*<br>*carA (EC:6.3.5.5),*<br>*carB (EC:6.3.5.5),*<br>*patB (EC:4.4.1.8),*<br>*sdaA (EC:4.3.1.17),*<br>*gadB (EC:4.1.1.15),*<br>*mtnE (EC:2.6.1),*<br>*dapA (EC:4.3.3.7),*<br>*dapB(EC:1.17.1.8)*	24<br>*lysA (EC:4.1.1.20),*<br>*dapF (EC:5.1.1.7),*<br>*dapE (EC:3.5.1.18),*<br>*dapH (EC:2.3.1.89),*<br>*(EC:2.6.1.17),*<br>*(EC:3.5.1.47),*<br>*murE (EC:6.3.2.13),*<br>*murF (EC:6.3.2.10),*<br>*carA (EC:6.3.5.5),*<br>*carB (EC:6.3.5.5),*<br>*patB (EC:4.4.1.8),*<br>*sdaA (EC:4.3.1.17),*<br>*gadB (EC:4.1.1.15),*<br>*mtnE (EC:2.6.1),*<br>*dapA (EC:4.3.3.7),*<br>*dapB(EC:1.17.1.8)*	21<br>*lysA (EC:4.1.1.20),*<br>*dapF (EC:5.1.1.7),*<br>*dapE (EC:3.5.1.18),*<br>*(EC:2.6.1.17),*<br>*murE (EC:6.3.2.13),*<br>*murF (EC:6.3.2.10),*<br>*patA (EC:2.6.1),*<br>*(EC:3.5.1.47),*<br>*lysC (EC:2.7.2.4),*<br>*dapA (EC:4.3.3.7),*<br>*dapH (EC:2.3.1.89),*<br>*gabD (EC:1.2.1.16*<br>*1.2.1.79 1.2.1.20),*<br>*dapB(EC:1.17.1.8)*	24<br>*lys*[illegible]<br>*dap*[illegible]<br>*dap*[illegible]<br>*(EC*[illegible]<br>*mu*[illegible]<br>*mu*[illegible]<br>*dap*[illegible]<br>*dap*[illegible]<br>*pat*[illegible]<br>*(EC*[illegible]<br>*LY*[illegible]<br>*gab*[illegible]<br>*1.2.*[illegible]
Arginine and proline metabolism	6<br>*pip (EC:3.4.11.5),*<br>*proB (EC:2.7.2.11)*<br>*proA (EC:1.2.1.41),*<br>*proC (EC:1.5.1.2)*	5<br>*pip (EC:3.4.11.5),*<br>*puuD (EC:3.5.1.94),*<br>*speG EC:2.3.1.57*	7<br>*pip (EC:3.4.11.5),*<br>*proA (EC:1.2.1.41),*<br>*proB (EC:2.7.2.11),*<br>*proC (EC:1.5.1.2)*	8<br>*pip*[illegible]<br>*pro*[illegible]<br>*pro*[illegible]<br>*pro*[illegible]<br>*cod*[illegible]

Classification	*L. plantarum* F42	*L. pentosus* P38	*L. argentoratensis* 15	*L. f*
				roc<br>*(EC*

Table S3 (*continued*)

Classification	*L. plantarum* F42	*L. pentosus* P38	*L. argentoratensis* 15	*L. f*
Histidine metabolism	11<br>*hisA (EC:5.3.1.16),*<br>*hisB (EC:4.2.1.19),*<br>*hisC (EC:2.6.1.9),*<br>*hisD (EC:1.1.1.23),*<br>*hisE (EC:3.6.1.31),*<br>*hisF (EC:4.1.3),*<br>*hisG (EC:2.4.2.17),*<br>*hisI (EC:3.5.4.19),*<br>*hisZ, (EC:3.1.3.15)*	1<br>*urdA (EC:1.3.99.33)*	11<br>*hisA (EC:5.3.1.16),*<br>*hisB (EC:4.2.1.19),*<br>*hisC (EC:2.6.1.9),*<br>*hisD (EC:1.1.1.23),*<br>*hisE (EC:3.6.1.31),*<br>*hisF (EC:4.1.3),*<br>*hisG (EC:2.4.2.17),*<br>*hisH (EC:2.4.2),*<br>*hisI (EC:3.5.4.19),*<br>*hisZ (EC:3.1.3.15)*	3<br>*his(*<br>*hisI*<br>*hisI*
Phenylalanine, tyrosine and tryptophan biosynthesis	23<br>*trpA (EC:4.2.1.20),*<br>*trpB (EC:4.2.1.20),*<br>*trpC (EC:4.1.1.48),*<br>*trpD (EC:2.4.2.18),*<br>*trpE (EC:4.1.3.27),*<br>*trpF (EC:5.3.1.24),*<br>*trpG (EC:4.1.3.27),*<br>*aroA (EC:2.5.1.54),*<br>*(EC:2.5.1.19),*<br>*aroB (EC:4.2.3.4),*<br>*aroE (EC:1.1.1.25),*<br>*aroC (EC:4.2.3.5),*<br>*aroK (EC:2.7.1.71),*<br>*aroD (EC:4.2.1.10),*<br>*tyrA2 (EC:1.3.1.12),*<br>*hisC (EC:2.6.1.9)*	4<br>*hipO (EC:3.5.1.32),*<br>*enr (EC:1.3.1.31),*<br>*dadA (EC:1.4.99.1),*<br>*(EC:2.5.1.19)*	21<br>*hisC (EC:2.6.1.9),*<br>*aroA (EC:2.5.1.54),*<br>*(EC:2.5.1.19),*<br>*aroC (EC:4.2.3.5),*<br>*aroE (EC:1.1.1.25),*<br>*aroD (EC:4.2.1.10),*<br>*aroK (EC:2.7.1.71),*<br>*trpA (EC:4.2.1.20),*<br>*trpB (EC:4.2.1.20),*<br>*trpC (EC:4.1.1.48),*<br>*trpD (EC:2.4.2.18),*<br>*trpE (EC:4.1.3.27),*<br>*trpF (EC:5.3.1.24),*<br>*trpG (EC:4.1.3.27),*<br>*tyrA2 (EC:1.3.1.12)*	14<br>*his(*<br>*paa*<br>*ato*<br>*aro*<br>*aro*<br>*(EC*<br>*(EC*<br>*aro*<br>*tyrA*

Table S4. The putative classification on the nucleotide metabolism. Enzymes were abbreviated and their information could be checked according to the EC number in KEGG database.

Classification	*L. plantarum* F42	*L. pentosus* P38	*L. argentoratensis* 15	*L. f*
De novo purine biosynthesis	11<br>*PRPS (EC:2.7.6.1),*<br>*purF (EC:2.4.2.14),*<br>*purD (EC:6.3.4.13),*<br>*purN (EC:2.1.2.2.),*<br>*purM (EC:6.3.3.1),*<br>*purK (EC:6.3.4.18),*<br>*purC (EC:6.3.2.6),*<br>*purB (EC:4.3.2.2),*<br>*purH (EC:2.1.2.3)*	13<br>*PRPS (EC:2.7.6.1),*<br>*purF (EC:2.4.2.14),*<br>*purD (EC:6.3.4.13),*<br>*purN (EC:2.1.2.2.),*<br>*purM (EC:6.3.3.1),*<br>*purK (EC:6.3.4.18),*<br>*purC (EC:6.3.2.6),*<br>*purB (EC:4.3.2.2),*<br>*purH (EC:2.1.2.3)*	11<br>*PRPS (EC:2.7.6.1),*<br>*purF (EC:2.4.2.14),*<br>*purD (EC:6.3.4.13),*<br>*purN (EC:2.1.2.2.),*<br>*purM (EC:6.3.3.1),*<br>*purK (EC:6.3.4.18),*<br>*purC (EC:6.3.2.6),*<br>*purB (EC:4.3.2.2),*<br>*purH (EC:2.1.2.3)*	9<br>*PRI*<br>*pur*<br>*pur*<br>*pur*<br>*pur*<br>*pur*<br>*pur(*<br>*pur*<br>*pur*
ATP and GTP metabolism	10<br>*purA (EC:6.3.4.4),*<br>*purB (EC:4.3.2.2),*<br>*guaC (EC:1.7.1.7),*<br>*guaB (EC:1.1.1.205),*	11<br>*purA (EC:6.3.4.4),*<br>*purB (EC:4.3.2.2),*<br>*guaC (EC:1.7.1.7),*<br>*guaB (EC:1.1.1.205),*	10<br>*purA (EC:6.3.4.4),*<br>*purB (EC:4.3.2.2),*<br>*guaC (EC:1.7.1.7),*<br>*guaB (EC:1.1.1.205),*	10<br>*pur*<br>*pur*<br>*gua*<br>*gua*

	guaA (EC:6.3.5.2), adk (EC:2.7.4.3), ndk (EC:2.7.4.6), gmk (EC:2.7.4.8)	*guaA (EC:6.3.5.2), adk (EC:2.7.4.3), ndk (EC:2.7.4.6), gmk (EC:2.7.4.8)*	*guaA (EC:6.3.5.2), adk (EC:2.7.4.3), ndk (EC:2.7.4.6), gmk (EC:2.7.4.8)*	[illegible]
Nucleoside metabolism	3	7	3	2
	iunH (EC:3.2.2.1), ade (EC:3.5.4.2)	*(EC:3.1.3.5), iunH (EC:3.2.2.1), ade (EC:3.5.4.2)*	*iunH (EC:3.2.2.1), ade (EC:3.5.4.2)*	[illegible]

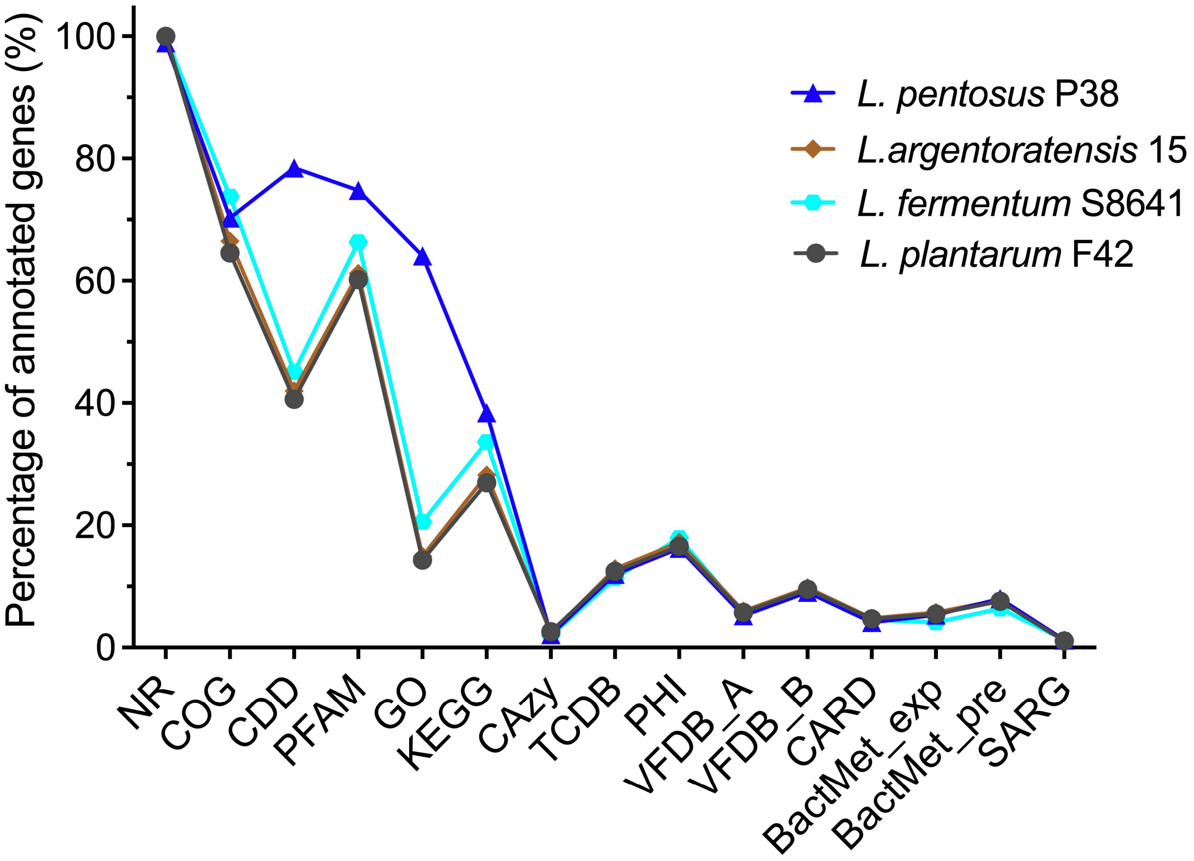

Figure S1. Percentage of annotated genes in different databases. NR (database of NCBI non-redundant protein sequences), COG (database of clusters of orthologous groups of proteins), CDD (conserved domain database), PFAM (protein family), GO (gene ontology), KEGG (kyoto encyclopedia of genes and genomes), CAZy (carbohydrate-active enzymes database), TCDB (transporter classification database), PHI (pathogen host interactions database), VFDB (virulence factors of pathogenic bacteria), CARD (the comprehensive antibiotic resistance database), BactMet (antibacterial biocide and metal resistance genes database), SARG (antibiotic-resistance gene database).

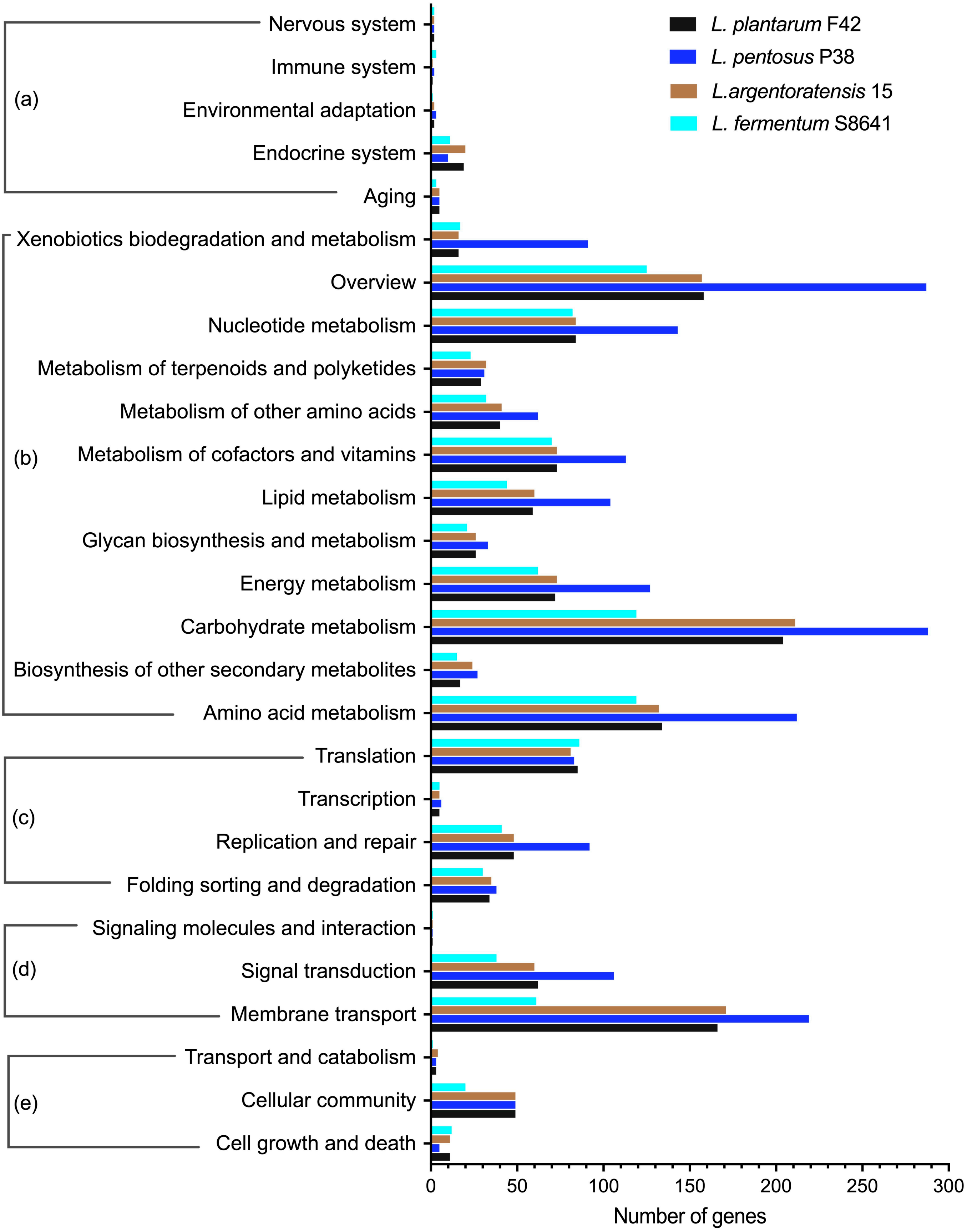
Nervous system
Immune system
Environmental adaptation
Endocrine system
Aging
Xenobiotics biodegradation and metabolism
Overview
Nucleotide metabolism
Metabolism of terpenoids and polyketides
Metabolism of other amino acids
Metabolism of cofactors and vitamins
Lipid metabolism
Glycan biosynthesis and metabolism
Energy metabolism
Carbohydrate metabolism
Biosynthesis of other secondary metabolites
Amino acid metabolism
Translation
Transcription
Replication and repair
Folding sorting and degradation
Signaling molecules and interaction
Signal transduction
Membrane transport
Transport and catabolism
Cellular community
Cell growth and death
(a)
(b)
(c)
(d)
(e)
L. plantarum F42
L. pentosus P38
L.argentoratensis 15
L. fermentum S8641
0
50
100
150
200
250
300
Number of genes

Figure S2. KEGG pathway annotated categories gene for different *Lactobacillus* species. (a) organismal systems, (b) metabolism, (c) genetic information processing, (d) genetic information processing, (e) cellular processes.

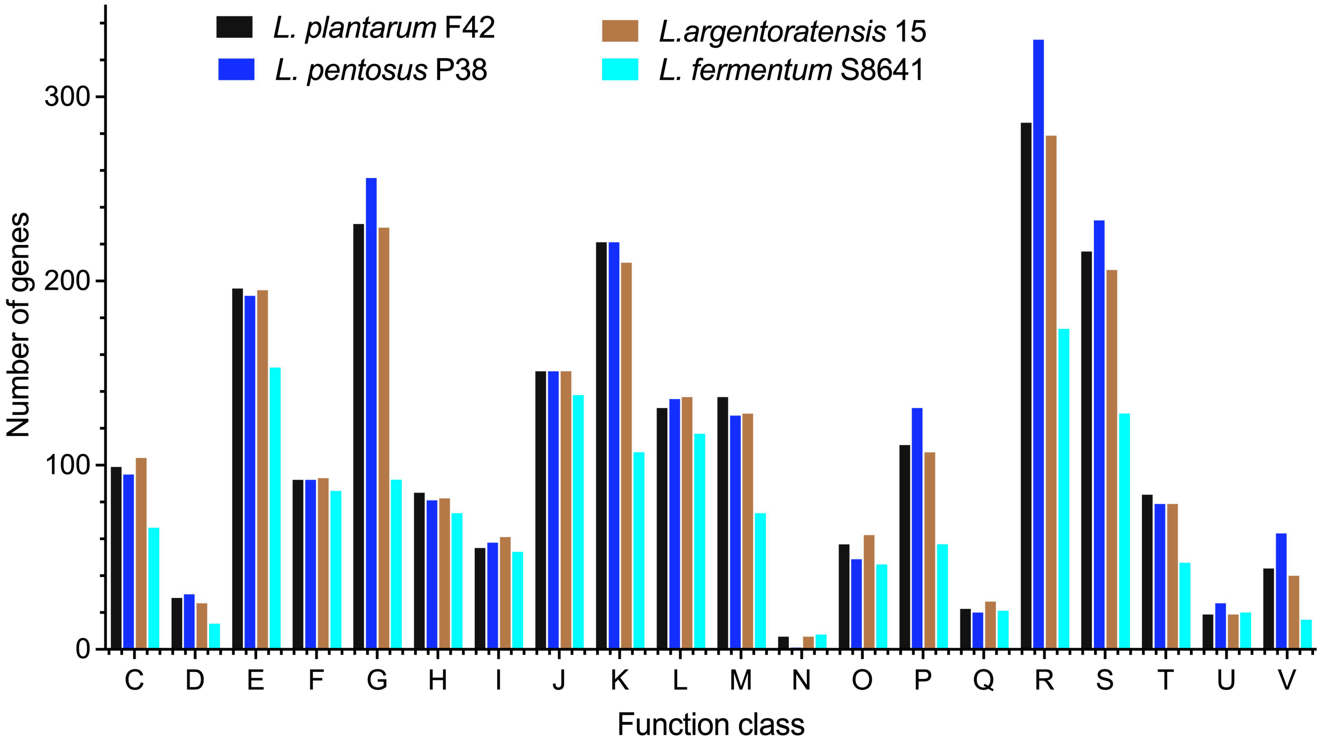

Figure S3. COG classification for *Lactobacillus*. A:RNA processing and modification, B:Chromatin structure and dynamics, C:Energy production and conversion, D:Cell cycle control, cell division, chromosome partitioning, E:Amino acid transport and metabolism, F:Nucleotide transport and metabolism, G:Carbohydrate transport and metabolism, H:Coenzyme transport and metabolism, I:Lipid transport and metabolism, J:Translation, ribosomal structure and biogenesis, K:Transcription, L:Replication, recombination and repair, M:Cell wall/membrane/envelope biogenesis, N:Cell motility, O:Posttranslational modification, protein turnover, chaperones, P:Inorganic ion transport and metabolism, Q:Secondary metabolites biosynthesis, transport and catabolism, R:General function prediction only, S:Function unknown, T:Signal transduction mechanisms, U:Intracellular trafficking, secretion, and vesicular transport, V:Defense mechanisms, W:Extracellular structures, Y:Nuclear structure, Z:Cytoskeleton.
